# Exercise Programs Combined with Diet Supplementation Improve Body Composition and Physical Function in Older Adults with Sarcopenia: A Systematic Review

**DOI:** 10.3390/nu15081998

**Published:** 2023-04-21

**Authors:** Marco Antonio Hernández-Lepe, Michelle Itzel Miranda-Gil, Edith Valbuena-Gregorio, Francisco Javier Olivas-Aguirre

**Affiliations:** 1 Medical and Psychology School, Autonomous University of Baja California, Tijuana 22390, Mexico; marco.antonio.hernandez.lepe@uabc.edu.mx; 2 Health Sciences Department, University of Sonora, Cajeme 85010, Mexico; a220223166@unison.mx (M.I.M.-G.); edith.valbuena@unison.mx (E.V.-G.)

**Keywords:** sarcopenia, exercise, physical activity, elderly, food supplementation, protein

## Abstract

Sarcopenia is a progressive and frequent syndrome among older adults highly related to physical inactivity and malnutrition. Nowadays, it is considered a pathology that triggers multiple health complications associated with the loss of muscle mass, strength, autonomy, and quality of life. The objective of the present systematic review was to evaluate the effect of exercise programs combined with dietary supplementation on body composition as the primary outcome. This systematic review was carried out in accordance with the elements considered for planning a systematic review by the Preferred Reporting Items for Systematic Reviews and Meta-Analyses (PRISMA), and the search was performed in the Scopus, EBSCO, and PubMed databases for the last 10 years. A total of 16 studies met the inclusion criteria and were included in this systematic review. Regular resistance exercise together with daily essential amino acids or whey protein and vitamin D supplementation improve the maintenance or gains in appendiceal/skeletal muscle mass and total lean mass in sarcopenic older adults. The data suggest a synergistic effect not only on the primary outcome, but also on other variables such as strength, speed, stability, and other indicators of quality of life. This systematic review was registered in PROSPERO, ID: CRD42022344284.

## 1. Introduction

Age-related muscle loss is a progressive and frequent phenomenon among older adults and is known as sarcopenia; nowadays, more than a syndrome it is considered a pathology which multiple health complications [1]. The definition of sarcopenia varies substantially depending on the study group that proposes the criteria for its diagnosis, although in general they include criteria such as low muscle mass, low muscle strength, and/or reduced physical performance. In this sense, the prevalence of this disease varies according to these diagnostic criteria; however, sarcopenia is estimated to affect about 10% of the over-65 population and increases to 30% in men over 80 years old. Moreover, this phenomenon will be complicated in the near future due to the increase in the population over 60 years of age, which is expected to reach about two billion by 2050 [2]. The most frequent complications associated with sarcopenia are less autonomy of the elderly regarding regular activities, loss of strength in the extremities, physical disability, poor quality life, and overall mortality [3,4].

Promotion of systematic physical exercise programs and following a healthy diet and/or supplementation are the most common strategies to prevent and treat sarcopenia in the elderly population, and even though there are many studies that carried out exercise programs or nutritional strategies against excessive body mass and physical function loss [5,6,7,8], only a few studies have been dedicated to analyzing the possible synergistic effects of these therapies on a regular basis and not only acutely [9,10]. In this sense, a problem arises for the holistic treatment of the pathology with multiple consequences.

In principle, it has been suggested that any type of physical activity contributes to improvements in the body and functional status of individuals. For example, Chen et al. [11] evaluated the effect of different types of exercise programs (resistance, aerobic, and combination training) on elderly population, concluding that all types of exercise programs increased the muscle mass and reduced the body fat percentage, with the most significant effects in the combination training group. It is necessary to mention that some reports clearly describe the metabolic adaptations (hormonal and other signaling molecules) related to the physical function and body composition emphasizing the influence exerted by resistance training on such markers as follistatin, myostatin, growth differentiation factors or stimulation of anabolic pathways [12,13].

Additionally, the effects of protein supplementation on body composition in the elderly are well-documented in the literature, with very different doses and a duration of between four and 36 weeks of supplementation, resulting in a reduced body fat mass and an increasing body lean mass, but the effects on physical function are not clear and need further investigation [14]. Another kind of supplements evaluated in the same age group resulted in the discovery of a beneficial effect on body composition of the intake of 3 g beta-hydroxy-beta-methylbutyrate [15] and 240 μg/day L-selenomethionine for 3 months [16]. Dietary supplementation culminates with the contribution of essential components for the maintenance of reserves, muscle recovery, or remodeling of muscle fibers. However, the results in the maintenance or gains in lean mass can be influenced by factors inherent to the type of physical activity, time, or intensity [17].

Not exploring both events together would reduce the possible synergistic effects of therapies that have been shown to be functional for the loss of lean mass. In this sense, the objective of the present review was to evaluate the effect of exercise together with dietary supplementation on body composition and physical functions as primary outcomes.

## 2. Materials and Methods

### 2.1. Study Search

This systematic review was carried out in accordance with the elements considered for planning a systematic review by the Preferred Reporting Items for Systematic Reviews and Meta-Analyses (PRISMA) website [18]. In addition, the systematic review protocol and considerations were registered in the International Prospective Register of Systematic Reviews (PROSPERO), ID: CRD42022344284.

The EBSCO, Scopus, and PubMed databases were employed for this research. The search strategy was delimited by the following descriptors and Boolean operators: (“Older adults” OR “Elderly”) AND “Supplementation” AND (“Physical Activity” OR “Exercise”) AND “Body composition” AND “Physical function”. The identified records were screened according to their publication date (within the last 10 years (January 2012–December 2022)).

### 2.2. Eligibility Criteria

The articles analyzed in this review include full texts of the randomized clinical trials (RCT) that evaluate the synergic effect of dietary supplementation and exercise or resistance training. The specific characteristics of the RCTs were determined according to the PICO strategy:

Participants: Healthy older adults of both genders aged 60 and over.

Intervention: Protocols that included a supplementation–exercise group with at least two sessions per week.

Comparison: RCTs with a comparison of a synergic treatment with a supplementation-only or an exercise-only group.

Outcomes: Studies that reported changes in body composition or improvements in physical components as their primary outcome were selected.

### 2.3. Exclusion Criteria

Articles with any of the following were excluded: duplicated, limitations in the design (less than 8 weeks of duration, for example) or implementation of the intervention, lack of control in the evaluations, inconsistency in the results.

### 2.4. Methodological Quality/Risk of Bias Assessment

The methodological quality of the selected studies (risk of bias) was assessed by screening the full texts and applying the Physiotherapy Evidence Database (PEDro) scale score [19]. Giving the value of 0 or 1 to a total of 10 items, the methodological quality score is defined as “poor” (total score under 4), “fair” (total score of 4 or 5), “good” (total score between 6 and 8), and “excellent” (total score of 9 or 10).

### 2.5. Data Analysis

To achieve the objective, the selected RCTs were summarized in a matrix that allowed the analysis of the main characteristics and outcomes.

## 3. Results

**Figure 1:**
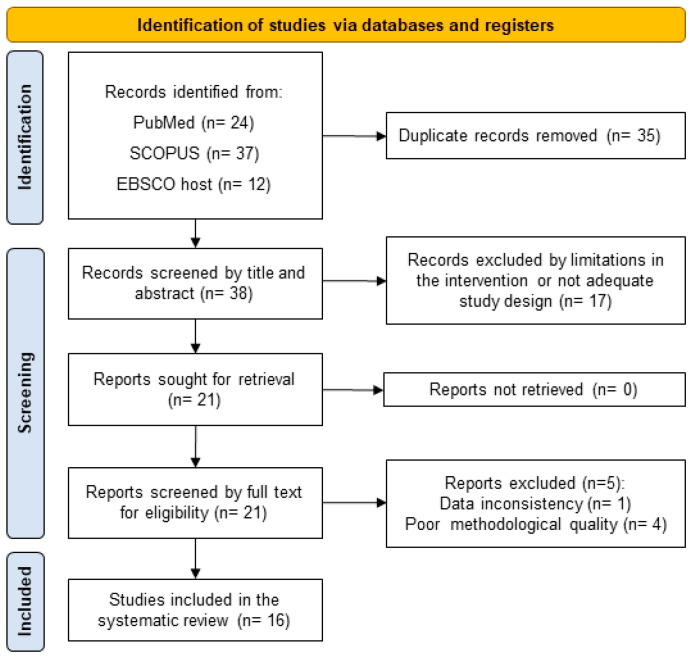
The PRISMA 2020 flow diagram for new systematic reviews.

The initial search strategy provided 73 results including the three databases mentioned above. After eliminating 35 duplicates, 38 RCTs were analyzed based on their abstracts and considering the exclusion criteria. A total of 18 studies were excluded for the short duration of the intervention, the inclusion of older adults with pathologies or non-specific supplements. A total of 20 studies met the inclusion criteria and were included in the methodological quality/risk of bias assessment, resulting in a total of 16 studies included in the present systematic review (Figure 1).

Regarding the methodological quality/risk of bias assessment, four studies were excluded for resulting in a PEDro scale score under seven (Table 1). A total of 16 studies met the inclusion criteria and were over seven in the PEDro scale score and were included in this systematic review, however, four of them had the minimum score of seven and two resulted in the scale score of 10 (excellent methodological quality).

Table 2 contains the included RCTs and their characteristics. The participants in each study included adults ≥ 60 years. A total of 1585 participants were analyzed together in the present review. Half of the studies evaluated protein supplementation from different sources with a particular trend towards whey protein. Six of the studies evaluated the influence of vitamin D, mostly in conjunction with amino acids (particularly leucine) or proteins. A few studies (two) evaluated the effect of non-nutrients (flavonoids (catechin)) in a liquid matrix. At the same time, the exercise applied in the evaluations included strengthening, bodyweight resistance and resistance band exercises, balance exercises, or aerobic training. All the interventions had a minimum duration of 8 weeks, although the range of these was between 8 and 40 weeks.

The included studies also evaluated other variables, such as biochemical markers and quality of life, the principal results of the effects (not specified, statically significant, nonsignificant improvement, or no effect) are described in Table 3, and a detailed explanation of them is provided in the following Results subsections.

### 3.1. Body Composition

Protein supplementation plus vitamin D was an effective strategy for maintaining or gaining muscle mass in older adults with sarcopenia. Of the studies analyzed that presented favorable results, six used supplementation with some type of proteins or essential amino acids [22,24,25,28,32,38], three—with vitamin D with or without proteins [23,26,37], and one—with catechin [31]. Yamada et al. [35] evaluated the effectiveness of 10 g whey protein plus 800 IU vitamin D on 112 older adults with sarcopenia. After 12 weeks of intervention, the control group lost 720 g of the appendiceal muscle mass, while the supplementation + exercise group maintained their muscle mass in the evaluated period. Beyond maintenance and in the same period, Rondanelli et al. [26] reported that moderate intensity training in conjunction with 22 g whey protein and 100 IU vitamin D was effective to obtain a net gain of 1.4 kg of fat-free mass in older adults.

It is important to note that medium-intensity or resistance exercise is by itself a determining factor for muscle maintenance or anabolism; however, the findings reported by several authors emphasized the additive effect of resistance training when accompanied by supplementation [9,10]. Supporting this statement, Miller et al. [39] reported a net gain of 140 g in lean appendicular mass in elderly adults supplemented with proteins + vitamin D (40 g and 2000 IU, respectively); however, these gains were 142% higher when supplementation was accompanied by progressive resistance training. The same phenomena were reported by Kim et al. [25] for training interventions combined with essential amino acids and by Zhu et al. [30] where the appendiceal mass of 76 older adults with sarcopenia increased thanks to resistance exercise and protein supplementation. In this last case, the additive benefits were four times greater in the supplementation + exercise group than in the placebo + exercise group.

In contrast, some authors suggest that exercise is primarily responsible for improvements in muscle maintenance or anabolism. In the studies by Griffen et al. [32] and Chalé et al. [33], whey protein supplementation had no additional effects compared to resistance training on fat-free mass, total lean mass, or skeletal muscle mass. For this, Grönstedt et al. [28] propose that adherence to the combined intervention (exercise + supplementation) plays an important role in the variables, since when individuals are classified based on the adherence to treatment, the fat-free mass gain is 63% higher in the high adherence group compared with those with low adherence.

### 3.2. Physical Functions

One third of the reports analyzed showed significant effects on the maintenance or gain of muscle strength during the combined intervention. Leg extension, leg press, back strength, or handgrip test were the primary outcomes improved. Although a few studies exclusively associated resistance exercise with strength gains [30,37], the effects were enhanced when accompanied by whey protein or essential amino acids [24,25,26,32,39]. It is necessary to point out that gains in strength were associated with the total skeletal muscle mass or the muscle mass of a specific limb, improving what researchers refer to as muscle quality [24,26].

Gait speed and distance traveled increased in the supplementation/exercise group greater than both in the supplementation group or the placebo group [32]. These benefits were reported not only for the usual walking speed, but also for the maximum walking speed, increased displacement speed (between 15 to 17%) based on the initial numbers [25,31]. Moreover, supplementation and exercise together also significantly improved coordination and gait stability. Compared to the exercise-only group, the results were 60% higher when exercise was accompanied by proteins and vitamin D as already described for other outcomes [39].

It is important to emphasize that although speed tests are evaluated at relatively short distances, the benefits that have been reported can be maintained at 400 m, thus demonstrating the resistance of the adaptations achieved to fatigue [24].

### 3.3. Biochemical Markers

The provision of supplements in general did not provide biochemical changes in sarcopenic elderly adults. This statement is generally supported since serum markers of malnutrition such as creatinine, prealbumin, or albumin remained unchanged [21,22]. While some researchers reported an improvement in the inflammatory status (such as an increase in plasma IL-10 [39] or a decrease in plasma IL-6 and TNF-α [32]), other authors found the opposite, thus presenting an unclear trend [21,27,28].

### 3.4. Life Quality

Only three of the studies analyzed report findings regarding improvement in the quality of life. Moreover, no statistically significant changes between the groups were identified in one of them. Only two reports [26,28] presented results referring to the improvement in health status (mental or physical abilities). Vitality and general health perception were the primary outcomes that improved after the intervention, while the caregiver time decreased significantly.

## 4. Discussion

In 1988, the definition of sarcopenia (from the Greek roots *sarx*—meat and *penia*—poverty, lack) was first established in order to explain the syndrome in which age favors a decrease in the lean body mass and, consequently, a deterioration of gait, mobility and, eventually, functional dependence in the elderly, and an increased morbidity and mortality risk [40]. The etiology of sarcopenia is the result of a combination of genetic, physiological, and environmental factors in which inadequate nutrition and physical inactivity are intimately related [41]. In this sense, exercise training or some dietary supplements seem to counteract age-related muscle loss. The objective of this systematic review was to examine the characteristics of exercise training-based programs (such as frequency, duration, or intensity) in conjunction with dietary supplements that have been reported to be effective in maintaining or gaining the lean body mass in older adults with sarcopenia.

In principle, high amounts of complete proteins are essential factors for muscle anabolism. Although some authors evaluated the potential of different compounds such as essential amino acids, BCAAs, milk fat globules, or catechin from tea, none of these proved to contribute favorably to the sarcopenia process. However, 80% of the articles that showed favorable changes in the muscle mass employed complete proteins (mainly milk derivatives (whey protein and caseinates)) [23,25,26,28], a minority (n = 1)—vitamin D [37]. It is well-accepted that the reduction of food intake with age contributes to a decrease in protein intake and consequently to a negative nitrogen balance, muscle atrophy, and aggravation of sarcopenia [41]. Therefore, supplementation with complete proteins proved to be effective in counteracting this cascade of events.

Complete proteins can provide essential amino acids to maintain muscle mass, but also signal cascades that promote its anabolism. Caseinates and whey proteins have been shown to have a complete aminogram that contributes “essential building blocks for anabolism” [42]; however, they also contain significant amounts of leucine which has been described to be an anabolic stimulus activating pathways such as mTOR and promoting muscle synthesis [43]. It is worth mentioning that some authors have proposed that sarcopenia is characterized by a resistance to the previously described anabolic stimulus. However, aerobic or resistance exercise has been described as being able to recover the lost sensitivity [44] and counteract this resistance. Thus, the recommendations of physical activity and protein supplementation were effective in the reports analyzed here.

Physical inactivity and short sun exposure in older adults contribute negatively to vitamin D synthesis. Recent findings indicate that vitamin D deficiency is highly prevalent (about 50.4%) in the elderly population worldwide [45]. In addition, it has been described that vitamin D by itself can regulate the expression of genes involved with muscle atrophy such as atrogin-1 and cathepsin L [46]. This justifies the rationale for why the multiple interventions listed in Table 1 employed vitamin D (in combination with proteins or alone) and showed muscular benefits in trained elderly people [23,26,37].

It is important to point out that although the mechanisms are well-described, some authors have evaluated protein or amino acid supplementation associated with some type of training without finding beneficial effects on sarcopenia, although many of them did not provide complete proteins [23,24,25,34,38], which would make adequate protein synthesis impossible, resulting in no statistical changes on muscle gain even with exercise training programs (at least two days/week for 12 to 24 weeks). Although it seems paradoxical, in reality, only a few of these authors actually monitored adherence to the proposed intervention in terms of physical activity and energy or proteins consumed (only two) [20,33]. When caloric or protein intake was monitored before and after the intervention, neither of these two indicated a change in either group (neither placebo nor exercise + supplementation) [20]. The data suggest that supplements are sometimes perceived as a replacement food because even when the intervention design “ensures” an increase in energy or protein intake, older adults reduce their usual food intake [20,33]. Grönstedt et al. [28] did consider stratifying subjects according to the adherence to treatment. In agreement with the assumption, the supplementation + exercise group with low adherence gained muscle mass, but the gain was 63% higher in the high-adherence group.

Although the primary findings on muscle maintenance or gain and physical abilities (speed, movement, coordination, etc.) indicate a clear picture, the secondary biochemical outcomes did not. Biochemical indicators were difficult to interpret due to interrelated issues. For example, although decreases in anti-inflammatory proteins such as IL-10 were reported [39], increases in proinflammatory proteins such as TNF-α were also reported [21,28]. In order to explain the phenomenon, it is necessary to understand the duality of regular exercise as a “stressor agent” but also as a “promoting factor” for weight loss. It is generally accepted that regular exercise sessions stimulate the release of inflammatory mediators such as cytokines [47]. On the other hand, exercise sessions have been shown to increase caloric expenditure and decrease the percentage of fat in individuals [48]. Thus, reduction in both the total fat mass and waist circumference would in turn promote changes in inflammatory markers, thus complicating the interpretation of the biochemical measurements reported [39].

One of the strongest limitations of the reports discussed is that some of them did not have the correct control, that is, they only considered a control group (without intervention) versus a group with supplementation and exercise; however, this type of design does not allow us to establish whether the findings generated were caused by additive effects or only one of the two factors was responsible for the changes reported. Furthermore, it is necessary to point out that the quality of life remains underestimated by the scientific community. In this sense, domains such as health perception, autonomy, positive attitude, and/or emotional comfort are underexplored variables highly related to the recovery of health in older adults with sarcopenia. These areas must be addressed in future research.

## 5. Conclusions

Resistance exercise (at least five times per week) together with daily essential amino acids (3 g) or whey protein (22–36 g) supplementation improves the maintenance or gains in the appendiceal/skeletal muscle mass and total lean mass in sarcopenic older adults. The data suggest a synergistic effect not only on the primary outcome, but also on other variables such as strength, speed, stability, and other indicators of the quality of life. In this sense, this strategy is suggested to limit the burden associated with musculoskeletal diseases and the individual and social complications associated with it. Moreover, future studies should focus their efforts on establishing optimized strategies based on their adherence to both resistance exercise programs and diet/supplementation recommendations to ensure the benefits discussed.

## Figures and Tables

**Table 1:** Physiotherapy Evidence Database scale score of the full texts of the screened studies.

Study	Internal Validity Items								Statistical Reporting Items		Total Score
	RA	CA	BC	BP	BR	BAD	AFU	ITTA	BGSC	RPM	
[20]	1	1	1	0	0	0	1	1	1	1	7
[21]	0	1	1	0	0	0	0	1	1	1	5
[22]	1	1	1	1	0	0	0	1	1	1	7
[23]	1	1	1	1	0	0	1	1	1	1	8
[24]	1	1	1	1	1	0	1	1	1	1	9
[25]	1	1	1	0	1	0	1	1	1	1	8
[26]	1	1	1	1	1	1	1	1	1	1	10
[27]	1	1	1	1	1	0	1	1	1	1	9
[28]	1	1	1	0	0	0	1	1	1	1	7
[29]	1	1	1	0	1	0	1	1	1	1	8
[30]	1	1	1	0	0	0	0	1	1	1	6
[31]	1	1	1	0	1	0	1	1	1	1	8
[32]	1	1	1	1	1	1	1	1	1	1	10
[33]	1	1	1	0	1	1	1	1	1	1	9
[34]	1	1	1	0	0	0	1	1	1	1	7
[35]	1	1	1	0	0	0	0	1	1	1	6
[36]	1	1	1	1	1	0	1	1	1	1	9
[37]	1	1	1	0	0	0	1	1	1	1	7
[38]	1	1	1	0	1	0	1	1	1	1	8
[39]	1	1	1	0	0	0	0	1	1	1	6

RA: random allocation; CA: concealed allocation; BC: baseline comparability; BP: blinding of participants; BR: blinding of researchers; BAD: blinding of analyst of data; AFU: adequate follow-up (>85%); ITTA: intention-to-treat analysis; BGSC: between-group statistical comparison; RPM: reporting of point measures and measures of variability.

**Table 2:** Randomized clinical trials that included exercise and supplementation as the strategy to improve sarcopenia in elderly adults.

Author, Year (Country)	n	Population (Age)	Type of Exercise (Frequency per Week)	Supplement Doses	Duration (Weeks)	Main Outcome
Mori and Tokuda, 2018 (Japan) [20]	75	Older women, 65–80 years	Bodyweight resistance and resistance band exercises (twice per week)	Whey protein, 25 g	24	Skeletal muscle mass index increased
Amasene et al., 2019 (Spain) [22]	28	Hospitalized patients, ≥70 years	Resistance training (twice per week)	Whey protein, 20 g	12	All the variables remained unchanged
Takeuchi et al., 2018 (Japan) [23]	63	Older adults, ≥65 years	Rehabilitation program and resistance training (NS)	BCAAs, 2.5 g Vitamin D, 12.5 μg	8	Handgrip strength increased significantly over time
Markofski et al., 2018 (United States of America) [24]	45	Older adults, ≥70 years	Aerobic exercise (three times per week)	EAAs, 15 g	24	VO_2_ peak and muscle quality increase in the exercise–supplementation group
Kim et al., 2012 (Japan) [25]	144	Older women, ≥75 years	Strengthening exercises and resistance band exercises (twice per week)	AA mix, 3 g	12	Walking speed, leg muscle mass, and knee extension strength increase in the exercise–supplementation group
Rondanelli et al., 2016 (Italy) [26]	130	Older adults, ≥65 years	Bodyweight resistance and resistance band exercises (five times per week)	Whey protein, 22 g Vitamin D, 2.5 µg	12	The supplementation group had a greater strength and muscle mass (1.6 kg)
Kim et al., 2015 (Japan) [27]	123	Older women, ≥75 years	Strengthening exercises and resistance band exercises (twice per week)	Milk fat globule membrane, 1 g	12	Walking speed and up-and-go time increase in all the groups
Grönstedt et al., 2020 (Sweden) [28]	102	Older adults, ≥75 years	Sit-to-stand exercises (daily)	Protein, 36 g	12	Significant increase in the body mass index in the exercise–supplementation group, with a tendency to increased lean mass (*p* = 0.30)
Unterberger et al., 2022 (Austria) [29]	116	Older adults, 65–85 years	Resistance training and weight exercises (twice per week)	Vegetal protein, 32 g	8	Physical function and muscle quality increase in the exercise–supplementation group
Kim et al., 2013 (Japan) [31]	116	Older women, ≥75 years	Stretching, muscle strengthening, balance, and gait training (twice per week)	Catechin, 540 mg	12	In the exercise–supplementation group, increased walking speed and decreased up-and-go time
Griffen et al., 2021 (United Kingdom) [32]	36	Older men, ≥66 years	Resistance exercises (twice per week)	Whey protein, 25 g	12	Leg press and gait speed increased while the fat mass percentage reduced
Chalé et al., 2012 (United States) [33]	75	Older adults, 70–85 years	Strength training exercises (three times per week)	Whey protein concentrate, 40 g	24	Total muscle cross-sectional area and lower extremity strength increased in all types of exercises in the exercise–supplementation group
Kim et al., 2016 (Japan) [34]	137	Older women, ≥70 years	Resistance and aerobic exercises (twice per week)	AA mix, 3 g Catechin, 540 mg	12	In the exercise–supplementation group, the number of steps and strides increased
Arnarson et al., 2013 (Iceland) [36]	141	Older adults, ≥65 years	Resistance training with a gradual increase in loads (three times per week)	Whey protein, 20 g	12	All the variables remained unchanged
Aoki et al., 2018 (Japan) [37]	130	Adults, ≥60 years	Single-leg standing and squatting (daily)	Vitamin D, 25 μg	24	Hip and knee strength increased
Kim et al., 2021 (Japan) [38]	124	Older adults, ≥65 years	Stretching, muscle strengthening, and gait training (once a week)	EAAs, 3 g	12	All the variables remained unchanged

NS: not specified; AA: amino acids; BCAAs: branched-chain amino acids; EAA: essential amino acids.

**Table 3:** Summary of the primary and secondary outcomes associated with exercise training and supplementation in elderly adults.

Author	*Body Composition*	*Physical Functions*		*Biochemical Analysis*	*Life Quality*
		*Strength*	*Gait/Walking Speed*		
Amanese et al., 2019 [22]	⊝	⊝	⊕	⊝	NS
Arnarson et al., 2013 [36]	⊝	⊝	⊝	NS	NS
Aoki et al., 2018 [37]	⊕	⊕	NS	NS	NS
Chalé et al., 2012 [33]	↔	↔	⊝	NS	NS
Griffen et al., 2021 [32]	↔	⊕	⊕	⊕	NS
Grönstedt et al., 2020 [28]	⊕	↔	⊝	⊝	⊕
Kim et al., 2012 [25]	⊕	⊕	⊕	NS	NS
Kim et al., 2013 [31]	⊝	↔	⊕	NS	NS
Kim et al., 2015 [27]	⊝	⊝	⊝	⊝	NS
Kim et al., 2016 [34]	⊝	⊝	⊝	↔	NS
Kim et al., 2021 [38]	⊝	⊝	⊕	NS	⊝
Markofski et al., 2018 [24]	⊝	⊕	⊕	NS	NS
Mori and Tokuda, 2018 [20]	↔	⊝	⊝	NS	NS
Rondanelli et al., 2016 [26]	⊕	⊕	NS	⊕	⊕
Takeuchi et al., 2018 [23]	⊕	↔	NS	NS	NS
Unterberger et al., 2022 [29]	⊝	⊝	⊝	NS	NS

NS: not specified; 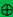: statically significant; 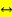: nonsignificant improvement; 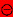: no effect.

## Data Availability

Data are available upon request to the first author (M.A.H.-L.).
